# Camouflage and Individual Variation in Shore Crabs (*Carcinus maenas*) from Different Habitats

**DOI:** 10.1371/journal.pone.0115586

**Published:** 2014-12-31

**Authors:** Martin Stevens, Alice E. Lown, Louisa E. Wood

**Affiliations:** Centre for Ecology and Conservation, College of Life and Environmental Sciences, University of Exeter, Penryn Campus, Penryn, Cornwall, United Kingdom; Evolutionary Biology Centre (EBC), Uppsala University, Sweden

## Abstract

Camouflage is widespread throughout the natural world and conceals animals from predators in a vast range of habitats. Because successful camouflage usually involves matching aspects of the background environment, species and populations should evolve appearances tuned to their local habitat, termed phenotype-environment associations. However, although this has been studied in various species, little work has objectively quantified the appearances of camouflaged animals from different habitats, or related this to factors such as ontogeny and individual variation. Here, we tested for phenotype-environment associations in the common shore crab (*Carcinus maenas*), a species highly variable in appearance and found in a wide range of habitats. We used field surveys and digital image analysis of the colors and patterns of crabs found in four locations around Cornwall in the UK to quantify how individuals vary with habitat (predominantly rockpool, mussel bed, and mudflat). We find that individuals from sites comprising different backgrounds show substantial differences in several aspects of color and pattern, and that this is also dependent on life stage (adult or juvenile). Furthermore, the level of individual variation is dependent on site and life stage, with juvenile crabs often more variable than adults, and individuals from more homogenous habitats less diverse. Ours is the most comprehensive study to date exploring phenotype-environment associations for camouflage and individual variation in a species, and we discuss the implications of our results in terms of the mechanisms and selection pressures that may drive this.

## Introduction

Animals show substantial variation in appearance among, and sometimes within species. The selection pressures underlying and driving this are varied, with multiple mechanisms often operating in the same population [1]–[5]. However, many animals apparently show clear links in form to their local environment, termed phenotype-environment associations. One of the most common instances for when this should arise involves camouflage, whereby individuals match the appearance of the environment or backgrounds against which they are found to avoid being detected or recognized by predators [6], leading to phenotype-environment matching. Camouflage is a longstanding textbook example of natural selection [7]–[9], yet relatively little work has involved quantifying the appearances of real species in the wild (but see for example [10], [11]). While substantial work in the last decade has been undertaken in artificial systems, using computer models, laboratory studies, or human made stimuli (e.g. [12]–[22]), our understanding of concealment in complex real-world habitats remains poor. Phenotype-environment associations for camouflage should involve the appearance of individuals being tuned to the features of their habitat. Specifically, we would expect individuals of different species that share the same backgrounds to converge in appearance, and individuals of the same species to become more dissimilar when they occupy different habitats.

Despite the above comparatively simple predictions, there remain substantial gaps regarding research on phenotype-environment associations and camouflage. Work to date has been largely restricted to investigating broad changes in overall body coloration without rigorous analyses of individual appearance, and has not investigated species with more complex patterns. For example, a recent study showed phenotype-environment associations in African desert jerboas (*Jaculus jaculus*) over large geographical scales, most likely driven by selection for camouflage from predators. This work showed correlations between the colors of individuals and predicted colors of the background from satellite images for where individuals were collected [23]. Although valuable there was, however, only basic assessment of the coloration of individuals from photographs, with red, green, and blue image values kept separate (i.e. not strictly speaking an analysis of color, which involves differences in reflectance in parts of a light spectrum). Other work on mice and reptiles has focused on understanding molecular mechanisms underlying different degrees of melanization (conferring body darkness), but rarely quantified individual appearance (e.g. [24]–[26]).

Phenotype-environment associations for camouflage are likely to occur frequently in invertebrates too, including commonly in marine environments. For example, the isopod *Idotea baltica* occurs in different color and pattern forms, with work showing that fish are more likely to attack less effectively camouflaged individuals, and there is some evidence for differences in morph frequencies associated with background types (albeit moderated by gene flow [27]). Such associations may be especially common in crabs. In India, the crab *Charybdis annulata* exists in at least two main color morphs (to human eyes), each associated with utilizing different substrates in their rocky shore habitat [28]. Other studies have also reported associations between phenotypes and habitats/backgrounds in a range of crab species, including changes in appearance with ontogeny, with such associations seemingly driven by a multiple mechanisms, including differential mortality, color change, decoration behavior, and phenotypic plasticity [29]–[31].

Perhaps the most in depth assessment of phenotype-environment associations for camouflage so far has been conducted in shore crabs (*Carcinus maenas*) in different habitats. Powell [32] found that crabs collected from South Wales were often patterned at small sizes (0-5 mm carapace width), and that with greater size the proportion of patterned crabs declined. Furthermore, he noted that larger crabs (>5 mm) seemed to vary in appearance depending on the habitat at the site from where they were collected. Hogarth [33] also collected juvenile crabs from different UK shore types and found that the level of pattern (described as high contrast, low contrast, or unpatterned) was higher when there was less fucoid algae and mud. More recently, and most comprehensively, Todd et al. [34] studied shore crabs at three study sites in Scotland representing a range of habitat types, including mussel beds, rockpools, seaweed, sandy beach, and rocks. They placed crabs into eight categories, largely based on factors such as the level of spotting and pattern types, and the amount of white, brown, or grey color, and tested whether morph proportions varied among sites. They found significant differences between sites with plain carapace crabs associated with macro-algal cover and patterned morphs associated with mussel beds. Todd et al. [35] then combined data from several studies investigating shore crabs at different spatial scales: micro (<1 m^2^), meso (100 s m^2^), and macro (10,000 s m^2^), and found evidence for associations between proportion of patterned and unpatterned crabs at each scale with different substrate types (e.g. amounts of mussel bed, rocks, and algae). The strongest associations were at the micro scale, with higher proportions of patterned crabs on mussel beds than on rocks or algae.

The studies above demonstrate how widespread and important phenotype-environment matching is likely to be in many species. However, as stated above, little work has objectively quantified appearance among individuals as opposed to human descriptions, and many studies of phenotype-environment associations involve species with simple colors and patterns. Thus, at present little work has directly quantified how specific features of color and pattern vary from one habitat or location to another. In addition, many species, including crabs, also show considerable individual variation at a given location, sometimes linked to ontogenetic changes [29]–[31]. This can either manifest as high continuous variation, or discrete morph types (e.g. polymorphism) [27], [33], [36]–[38]. In camouflage, such high variation could be driven by a wide range of processes, such as matching of individuals to different substrate patches, or polymorphism to defeat predator search images (e.g. [5], [12], [36]–[40]). However, little work has directly quantified the nature of this phenotypic variation in real animals, or related this to differences in habitats.

**Figure 1:**
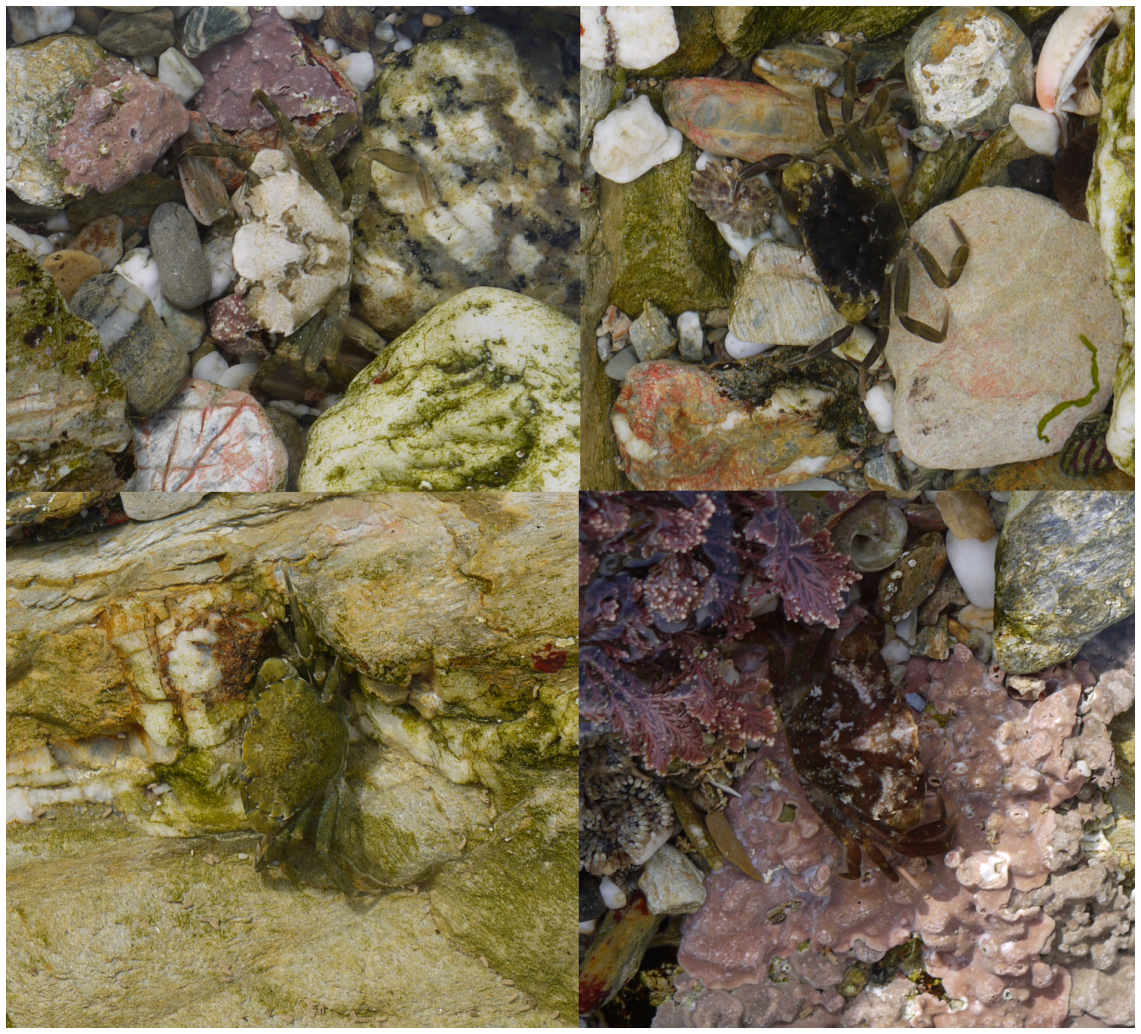
Examples of the camouflage of shore crabs with different colors and patterns. These crabs were all photographed at one of the study sites (Falmouth) and show how well different phenotypes can match different substrate patches.

**Figure 2:**
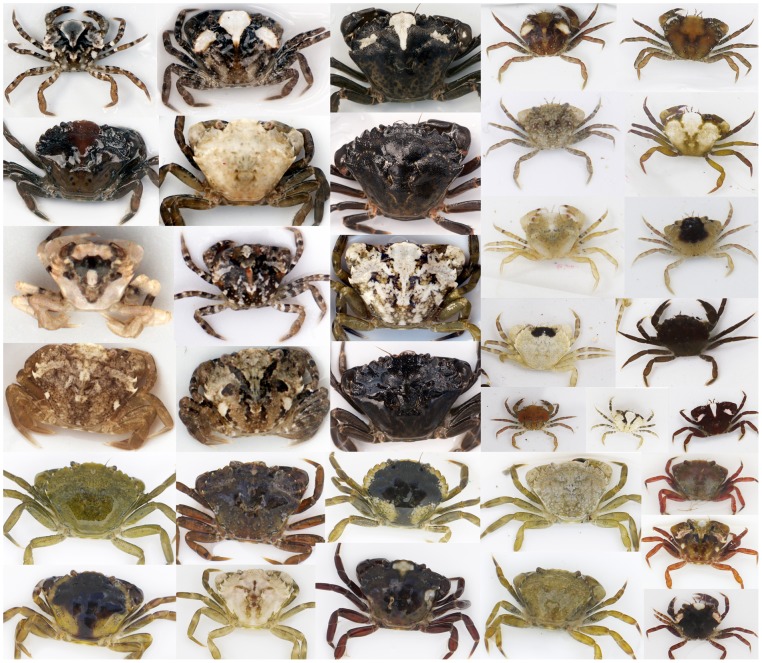
Variation in the colors and patterns of crabs. Shore crabs are highly variable in appearance, as demonstrated by the diversity of crabs in this image with the majority of them here collected from just one site (Falmouth). Crabs in the two right columns are mainly juveniles of sizes below approximately 10–15 mm carapace width, whereas crabs on the left three columns are individuals greater than 15 mm carapace width. Note that the crabs in this image show the range and extremity of forms that occur, rather than being representative of the most common phenotypes at any given site.

Here, we test for phenotype-environment associations in the appearances of the common shore crab from different habitats around Cornwall in the UK, and determine how crab colors and patterns vary with location. Furthermore, we quantify how such differences depend on life stage (adult or juvenile), and the level of inter-individual variation in appearance and how this relates to habitat. To human eyes, crabs are often extremely well camouflaged (Fig. 1), and the previous studies of Powell [32], Hogarth [33], and especially Todd et al. [34], [35] provide earlier evidence for phenotype-environment associations in this species. However, with the exception of quantifying the amount of white pattern on crabs via image analysis [34], [35], this work was based on human assessment of crab appearance. Furthermore, crabs were grouped into categories by eye, yet it is not clear that they exist in discrete morphs. Variation in crabs may instead be continuous, or at least seems to be extremely high, especially for juveniles (Fig. 2).

Shore crabs are an ideal species to address these issues, being the most widespread crab species in the UK and much of Europe, and found in a wide range of habitats (estuaries and mud flats, sandy beaches, pebbles, mussel beds, and rockpools) each with different appearances [41], [42]. They are also highly adaptable and an invasive species in many parts of the world [43], [44]. Carapace color is well known to vary in adults, but to human eyes is especially diverse in juveniles [33], [45]. It is widely reported that crabs differ in appearance with age, with crab patterns partially or fully disappearing with increasing age or size [33], [34], [42], [46], although this has not been systematically quantified. Both adult and juvenile crabs have a range of predators, including many bird species, especially gulls and shore birds, plus various species of fish [42], and so selection from visually guided predators is severe.

## Methods

### Study sites

We used four locations to sample crabs from around the Cornish peninsular in the southwest UK, which differed in the predominant habitat type. Site 1 was Falmouth (5°4′5"W, 50°8′41"N) on the south coast, comprising a stretch of shoreline collectively encompassing Castle and Gyllyngvase beaches. The site primarily comprises large areas of rockpools and rocky crevices with stony/gravel substrates in the pools, and lower down the rockpools increasing amounts of seaweed. Site 2 was Godrevy Point (5°23′33″W, 50°14′17″N) on the north coast, which primarily consisted of exposed rocky outcrops with mussel beds. Site 3 was Helford Passage, an estuarine location on the south coast (5°7′41″W, 50°5′59″N) comprising a large mudflat area, as well as some tiered craggy rock pools. Finally, site 4 was Summers beach at St Mawes (5°01.149″W, 50°9.445″N), also on the south coast, and comprising rockpools, gravel, and some low seaweed cover adjacent to a pebbled beach.

### Data collection

We conducted sampling at each site over three broad time periods to be sure that our sampling was not restricted to one interval that may be affected by changes in crab abundance or distribution (though we note that both adult and juvenile crabs are easy to find all year round in the region). Sampling was conducted in 2013 with periods broadly being mid April to early May, late May to early June, and mid August to early September, although owing to weather and tides some sampling days were somewhat later. We visited each site three times on different dates, roughly separated into spring, early summer and late summer. For each site and for any given sampling period we conducted one transect at three different heights on the shore (i.e. three transects in total per period). Originally, we split Falmouth into two areas (Gyllyngvase and Castle) as these are separated by a small sandy beach, and therefore undertook three periods of sampling at each place. However, this area essentially forms one almost continuous area of rockpools along a small stretch of coastline, and so we decided it was more appropriate to pool this dataset into one location. As such, we undertook twice as many transects at Falmouth than for the other sites, and partly for this reason this area has a larger sample size than the other sites. All work was conducted under approval from the University of Exeter Biosciences ethics committee (application 2013/75). The field locations (specified above) are publically accessible land and no further licenses or permits were needed. Crabs were kept no longer than 3 hours, and all individuals were returned unharmed to their original rockpool area after being tested. Shore crabs are not an endangered or protected species.

On each sampling event a 50 m (30 m at Godrevy point due to the nature of the beach) transect was placed horizontal to the shoreline corresponding to low, middle and high tide zones spaced evenly down the beach. Transect positioning was determined by measuring the depth of the beach and dividing into four sections. The upper transect was positioned approximately one quarter of the distance from the top of the mature rockpool area and the lower transect one quarter of the distance from low tide, with the middle transect positioned central to this. The approach was taken to ensure sampling on different heights of the beach while avoiding extreme low and high tide areas. Each transect was subdivided into 2.5 m intervals, whereby at each point a 0.5 m by 0.5 m quadrat was placed on either side of the transect. Each quadrat was then searched for shore crabs for a time period of five minutes (less if the sampling area was clearly exhausted before this time, such as in areas of bare rock). During the search, movable boulders, rocks and seaweed were turned over, crevices checked, and sand, mud and gravel raked using fingers in order to ensure crabs were unlikely to be missed.

After collection crabs were placed in buckets (white for sample period 1 and grey sample periods 2 and 3). Crabs were sexed according to their abdomen [41] and then photographed in human visible and ultraviolet (UV) light in a shallow grey tray next to a ruler (see below for photography and image analysis details). A black and silver photographic umbrella (Neewer Technology Ltd., Guangdong, China) was used to shade individuals from direct sunlight in order to reduce specular reflectance (where the light ‘bounces’ back off the crab carapace), and carapaces were gently dabbed dry with a cloth to remove surface water. Carapace width was taken from the digital photographs using the ruler included in the photos.

### Quantification of habitat composition

Although the field locations were quite different in appearance to us, we sought to quantify the proportion of different substrate types found in each site. To do so, we ran a further nine transects of 30 m at each site (three at each shore height). At each 1 m interval we simply recorded the substrate type present. From this, for each site and shore height we calculated the proportion of each background type present, allowing us to determine the difference in habitat composition between locations and to relate this to differences in crab appearance. We undertook these transects in May 2014. Although this was not the same year as when the crab sampling took place, we could detect no major changes in the appearance or composition of the habitats between years, meaning that any error is unlikely to be substantial.

### Image analysis and metrics of color and pattern

Photography and initial image calibration and analysis generally followed our previous recent methods [45]. Imaging was undertaken with two Nikon D7000 digital cameras, each of which had undergone a quartz conversion to enable UV sensitivity (Advanced Camera Services, Norfolk, UK), and fitted with a Nikon 105 mm lens. For the human visible photos, we placed a UV and infrared (IR) blocking filter in front of the lens, which transmits wavelengths only between 400–700 nm (Baader UV/IR Cut Filter). For the UV images, a UV pass and IR blocking filter was used (Baader U filter), which transmits between 300–400 nm. We have previously characterized the spectral sensitivity of our cameras (see [45]), in combination with the lens and filters, and their overall sensitivity for each image channel is approximately: UV: 360–400 nm (peak 380 nm), SW: 400–550 nm (peak 460 nm), MW: 420–620 nm (peak 540 nm), LW: 560–700 nm (peak 625 nm). For calibration purposes, each crab was photographed alongside a Spectralon grey reflectance standard (Labsphere, Congleton, UK), which reflects light equally at 40% between 300 and 750 nm. Images were taken in RAW format with manual white balance and a fixed aperture setting.

During calibration (undertaken in custom programs in Image J), images were converted to uncompressed TIFF files and the images of each crab comprised four bandpass layers corresponding to the long-wavelength (LW), medium-wavelength (MW), short-wavelength (SW), and UV parts of the spectrum. Most cameras show nonlinear responses in image value to changes in light levels that need to be corrected before accurate data can be obtained, and so we linearized all images based on quantified camera responses to a set of eight Spectralon grey standards with reflectance values ranging from 2–99% [47]–[49]. Next, we controlled for differences in ambient light conditions by standardizing (equalizing) the images to the grey standard, and scaled each image channel to reflectance, where an image value of 255 on an 8-bit scale equals 100% reflectance [47]. These images therefore corresponded to the physical reflectance properties of the crab patterns in four parts of the spectrum and could be used for analysis of coloration and pattern.

Following calibration, each image was first resized based on the ruler in the image so that all crabs were on the same scale for the pattern analysis. Then, for each of the four images we selected measurement areas (regions of interest; ROIs) of the entire crab carapace pattern, but avoiding any clear areas of specular reflectance. We then calculated several metrics of appearance based on color, brightness, and pattern. Brightness was calculated as (LW+MW+SW+UV)/4, and is a measure of how bright or dark crabs are across the entire spectrum [45], [50]. Although actual mechanisms of lightness perception in animals is normally based on one or two receptor types and often largely restricted to the middle part of the visible spectrum (medium wavelengths) [51] our aim here (as with [45], [50]) is to simply quantify the brightness of crabs overall without making assumptions about receiver vision, especially given the range of predators and visual systems that attack shore crabs [42]. An alternative approach is to base measurements of brightness on only the mediumwave (‘green’) part of the spectrum. However, this is unlikely to make any difference to our findings because in our dataset there is a very high level of correlation (R^2^ = 0.98) between crab brightness values based on mediumwave only and overall reflectance based metrics (see also [50]).

Next, we calculated two types of color metric for each crab, being saturation and hue. Saturation, generally considered as the amount of a given color compared to white light (i.e. how rich or deep the color is), can be calculated by plotting the position of each color in a tetrahedral space based on XYZ coordinates derived from the standardized (to a proportion of the total, to remove absolute brightness variation) LW, MW, SW, and UV values [52], [53]. Saturation can then be considered as the shortest distance from the achromatic center of the space to that color, with larger values suggesting greater saturation [53]. For hue, we followed previous approaches in basing measures on a ratio of reflectance in different parts of the spectrum [45], [54], [55]. Broadly, the rationale for this approach is based on how color in visual systems is encoded by opponent color channels, usually constructed as a ratio that defines the type of color present (e.g. red versus green: LW/MW) (see [45], [54]–[56] for more on the rationale and earlier uses of similar approaches). In our case, because we are not modeling actual visual pathways and because there is no *a priori* reason to define a specific color channel, we followed earlier approaches in using a principal component analysis (PCA) to determine the main axis of color variation that exists in the crabs, and to use this to inform our decision about a logical color channel(s) to use [45], [55]. We undertook a PCA on a covariance matrix of the standardized (proportional) LW, MW, SW, and UV channel data for all crabs measured, and used the resultant principal components (PCs) to determine color channels that best define the color variation present. Two principal components (PCs) explained 94.2% of the total variance (PC1 = 75.8%). These gave two color channels with Hue 1 being calculated as UV/((LW+MW+SW)/3), and Hue 2 as LW+UV/MW+SW. Hue 1 is essentially a ratio of UV reflectance to other parts of the spectrum, whereas hue 2 is a ratio of longwave (red or brown colors) and UV reflectance compared to mediumwave light (blue and green colors). We checked for correlations between our metrics of color and pattern, and found correlations between saturation and hue 1. We therefore conducted analyses with the following color metrics only: brightness, saturation, and hue 2 (henceforth just ‘hue’).

Shore crabs also differ greatly in pattern as well as color, and so we quantified the key features of crab patterns using a ‘granularity’ analysis approach. This has previously been used to analyze both the camouflage of cuttlefish patterns (e.g. [57], [58]), and bird egg markings (e.g. [59]). The technique involves decomposing an image into a series of different spatial frequencies (‘granularity bands’) using Fourier analysis and bandpass filtering, followed by determining the relative contribution of different marking sizes to the overall pattern. The filtering into different frequency bands functions like a sieve, capturing information at different spatial scales corresponding to different sized markings. Pattern analysis was conducted in custom files for Image J, with analysis based on different pixel sizes. That is, the analysis calculates the amount of information, or energy, corresponding to markings of different sizes, starting with small markings (few pixels) and increasing in size to larger markings. A log scale arrangement was used starting at two pixels for the smallest size and increasing with a log multiplier of 1.414 up to a maximum of 4096. This upper limit was chosen after a small subsection of crabs of a variety of size and pattern type were processed at a linear scale from two pixels in increments of 100 up to 16002 with little pattern detected over 4000 pixels. Restricting the number of size bands measured was necessary as greater numbers of bands substantially increases processing time.

For each granularity band, we calculated the overall pattern ‘energy’, being the sum of the squared pixel values in each image divided by the total number of pixels [57], [59], [60]. The energy values across all filtered images produce a ‘granularity spectrum’, being a plot of energy versus pixels (marking size). Note that this is the opposite to most previous work whereby energy is reported in terms of spatial frequency (with low spatial frequencies corresponding to large markings). Next, from each granularity spectrum we can calculate a range of information about crab patterns [59]. First, the maximum energy value at any point in the spectrum corresponds to the dominant marking size, allowing us to calculate the main marking size present in the pattern. Next, the proportion of the total energy across the entire spectrum corresponding to the maximum energy point provides a measure of pattern variation. A high value indicates that the pattern is dominated by one or a few marking sizes. Finally, the amplitude or total energy of the spectrum provides a measure of marking contrast [60], whereby higher values indicate greater contrasting markings. Thus, from this analysis we gained measurements of marking size, total energy (pattern contrast), and proportion energy (marking size variation).

Therefore, overall we calculated six different measures of crab appearance from our image analysis techniques: brightness (overall reflectance), saturation (color richness, e.g. red versus pink), hue (color type, red and ultraviolet versus blue-green color), proportion energy (how much one marking size dominates, or the diversity of marking sizes), total energy (amplitude, or pattern contrast), and marking size (the dominant marking size on the crab pattern).

### Diversity in appearance of crabs within and among sites

We also quantified the amount of variation that exists in both adult and juvenile crabs at each location. To do so, we utilized an approach previously developed to quantify polymorphism in the egg colors and patterns of parasitic and host birds in a ‘multidimensional phenotypic space’ (MDPS) [55]. This approach calculates the difference in appearance of pairs of crabs in a six-dimensional space, whereby each individual is defined by its measurements of hue, saturation, brightness, marking size, total energy, and proportion energy. First, each of the six variables was standardized to the same scale as one another in terms of a proportion of the maximum value for that variable for the entire dataset (i.e., all variables were on a scale between 0 and 1 [55]). Then, to calculate diversity in appearance in each data set (for each site and life stage), we first calculated the shortest (Euclidian) pairwise distances between each crab and every other crab in the phenotypic space. This yielded a matrix of distances for each crab versus all other crabs, of which we could then take the average for each crab. Larger distances mean that crabs are more spread out in the MDPS, and therefore have greater variability. However, because larger sample sizes may yield greater measures of diversity simply due to more individuals, we also conducted random resampling analyses to control for this effect [55]. To do so, we took 99 random samples of each dataset of the same sample size as the smallest dataset for a given comparison (e.g. for adults and juveniles at each site) and calculated the MDPS for each of these, followed by taking the average of each of these resampled datasets. We then compared these values (and their standard deviation) to the non-resampled datasets to determine the effect of different sample sizes. These analyses were undertaken using custom programs in MATLAB (Mathworks).

### Classification of size/age groups

In order to compare adult and juvenile crabs, we needed to be able to classify crabs into one age group or another. Sexual maturity in crabs can vary with size and location, and so initially we used two criteria to classify individuals based on previous work and our own initial studies, whereby the point of maturity is determined in relation to size. First, crabs were separated based on estimates of maturity taken in males and females [41], with maturity in females often reached by 28 mm CW, and in males above 21 mm, with individuals below these sizes often immature [61]. Hence, individuals were classified as mature or immature. Second, we classified crabs as adults when they were above 25 mm CW (regardless of sex), with individuals below 25 mm as juvenile. This size boundary was taken partly with regards to including some of the smaller range of sizes at which crabs will often become mature, and because past work has suggested that patterned crabs are consistently rare above 20–25 mm in carapace width [32], [33]. We then compared how effectively individuals fell into these categories based on our appearance metrics (brightness, hue, saturation, marking size, total energy, and proportion energy), using Discriminant Function Analysis (DFA) to decide which classification criteria to use in the other analyses.

### Statistics

We first used DFA to determine how effectively crabs were separated into our two potential age categories based on our appearance metrics (see above). For this we used the ‘lda’ function from the ‘MASS’ library in R [62]. We used a leave-one-out cross-validation (‘jackknifed’) DFA technique, which works by excluding one observation and formulating a discriminant function using the remaining data, followed by using that function to classify the excluded observation. This approach means that the classification is independent of the excluded sample, and the model is therefore more conservative than regular DFA. Our tests showed that crabs were most effectively classified based on defining adults as 25 mm CW or above, and juveniles as below 25 mm (see Results). Next, we conducted Spearman's rank correlation co-efficient analyses of crabs for each appearance metric versus continuous variation in size, to determine how crabs change appearance as they grow larger/older.

To determine how crabs differ in appearance with location, we ran General Linear Models (GLMs) on each appearance trait (e.g. brightness, marking size, and so on) as the response, with factors site (field location), shore height (low, middle, and upper zones), and life stage (adult or juvenile) as fixed effects. We also included interactions between site and height (to determine if changes in appearance with site depend on location on the shore), site and life stage (to determine if differences among site depend on adult or juvenile crabs), and height and life stage. Any non-significant interactions were removed from the model in a stepwise manner and the model rerun. Data generally needed transformation (specified in the Results) before it met the assumptions of GLM tests. We also performed a GLM to determine if crab sizes (response) differed with height on the shore and location.

Next, we aimed to determine how distinct crabs are from each location, and the degree of phenotypic overlap. To do so, we conducted DFA (separately on adults and juveniles) including our appearance metrics defined above, to determine the level of overlap in crab appearances between each site and the proportion of crabs misclassified into the wrong site category. As above, we used a leave-one-out cross-validation DFA technique for this.

Finally, for the MDPS variability data, we ran a GLM with MDPS values as the response, with site, life stage, and an interaction between site and life stage included in the model to test whether crabs from some locations are more variable than crabs from other sites, and whether this depends on age class (adult or juvenile).

## Results

We sampled 677 crabs in total from four sites of different habitat composition (see below): 264 from Falmouth (193 juveniles, 71 adults), 92 from Godrevy (63 juveniles, 29 adults), 158 from Helford (116 juveniles, 42 adults), and 163 from St Mawes (136 juveniles, 27 adults).

### Distinctiveness of adult and juvenile appearance

We used discriminant function analyses (DFA) to determine how effectively different life stages fall into predefined age groups, using the predictors of brightness, saturation, hue, total energy, proportion energy, and marking size. First, breaking down crabs into mature (n = 188) and immature (n = 489) for males and females, the proportion of correct classifications was to 0.790. Here, mature individuals are correctly assigned at a proportion of 0.423 (108 individuals misclassified as immature), whereas immature crabs are correctly classified at a proportion of 0.930 (34 individuals misclassified as adults). Second, breaking down crabs into juveniles (508) as individuals with carapace widths of <25 mm and adults (169) as crabs larger than this, produced a proportion of 0.836 crabs correctly assigned to their group. Adult crabs were correctly assigned at a proportion of 0.490 (87 individuals misclassified as juveniles), and juveniles were correctly classified at a proportion of 0.953 (24 misclassified as adults). Therefore, the second analysis best separates crabs into adults and juveniles with least overlap between categories, and so we used these categories in the further analyses.

### Relationships between size and appearance

**Figure 3:**
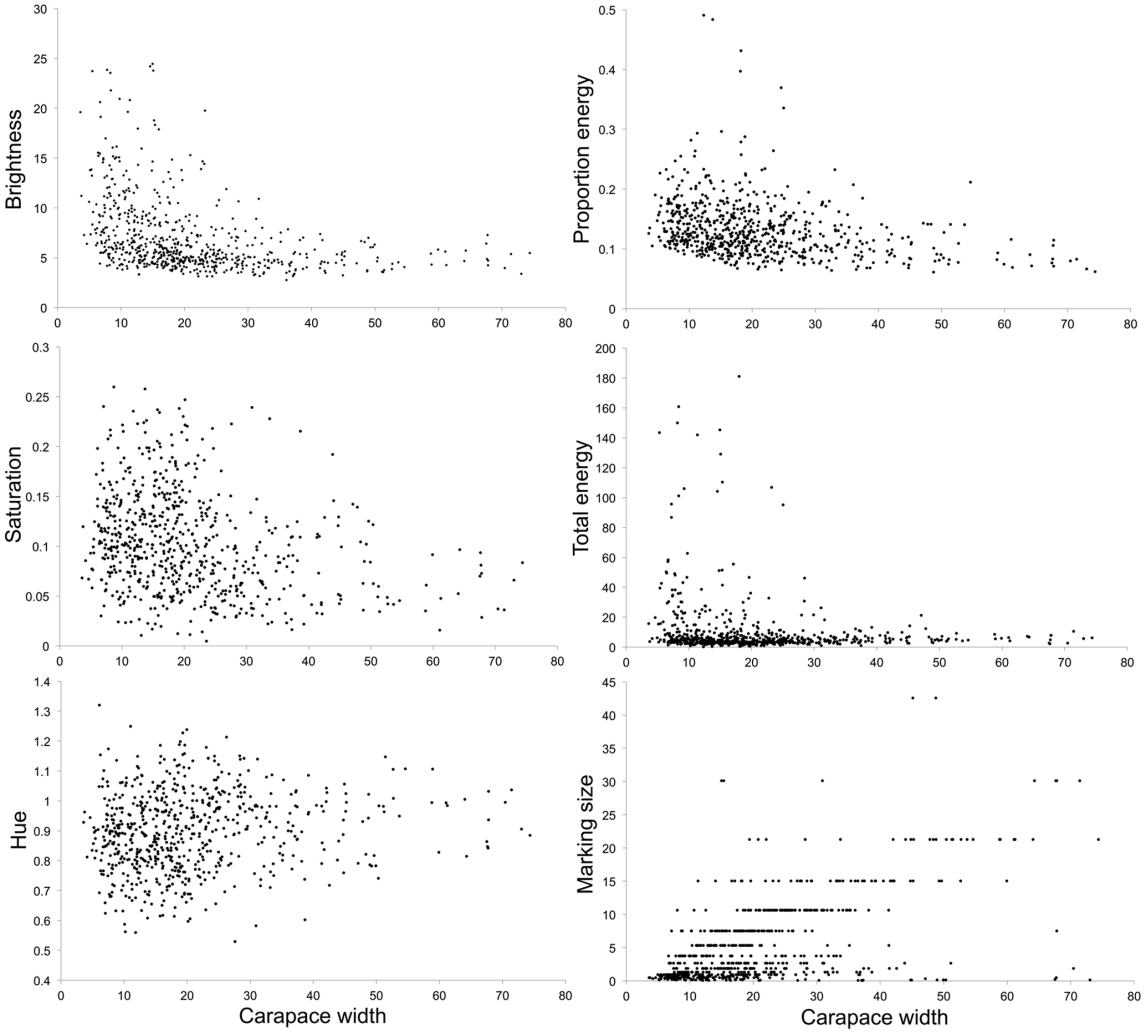
Correlation plots of the six different metrics of appearance with increasing crab size. All correlations were significant showing that crabs change in appearance as they grow larger, presumably through successive molts. For most variables the amount of spread in the data decreases with increasing crab size, showing that smaller crabs have high individual variation and larger crabs are more uniform.

There were significant relationships with all appearance metrics (Fig. 3). Larger crabs tend to be darker (lower brightness; N = 677, r_s_ = −0. 496, t = −14.84, df = 675, p<0.001), and have less saturated colors (N = 677, r_s_ = −0.284, t = −7.71, df = 675, p<0.001). Larger crabs also have less contrasting carapace patterns (lower total energy values; N = 677, r_s_ = −0.148, t = −3.89, df = 675, p<0.001), though unsurprisingly bigger crabs have larger markings (N = 677, r_s_ = 0.514, t = 15.58, df = 675, p<0.001). In addition, crabs have higher hue values as they get bigger (N = 677, r_s_ = 0.159, t = 4.20, df = 675, p<0.001). Here, crabs are moving closer to hue values of 1.00 as they get larger, essentially moving towards the achromatic point (i.e. becoming more grey/less blue-green in color). Note also that as crab sizes get larger the spread of values decreases, reflecting the fact that juvenile crabs show greater variation in appearance. Finally, crabs have lower proportion energy values when they are bigger, implying a larger range of marking sizes and less prominent markings (N = 677, r_s_ = −0.339, t = −9.37, df = 675, p<0.001).

### Differences in habitat composition among sites

**Figure 4:**
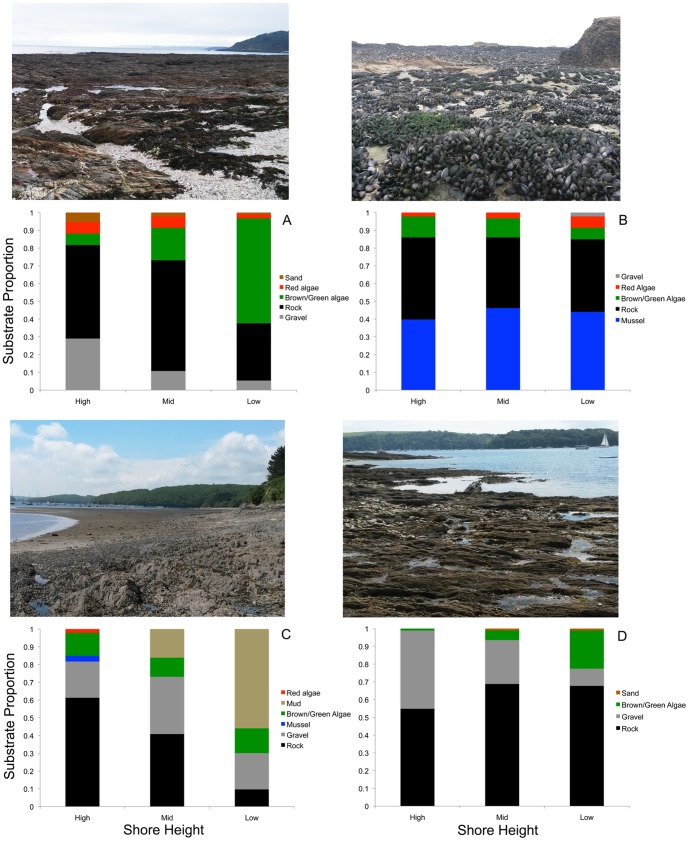
Proportions of different substrate types present at the four study sites. A. Falmouth is characterized by a range of substrates but predominately rock, gravel/stones, and green/brown seaweed, the latter of which increases greatly lower down the shore. B. Godrevy is a site dominated by mussel beds and rock with little change with shore height. C. Helford is a mudflat area with rockpools and gravel at high shore heights being replaced by flat mudflats lower down the shore. Note that even though the high tide zone is dominated by rock and gravel there is a great deal of muddy sediment here making the appearance at all three zones muddy. D. St Mawes is a rockpool site dominated by rocks and gravel substrate, with some green/brown algae especially lower down the shore. Although the number of substrate types present is low, subjectively the appearance of the substrate is very diverse in color and contrast. Images show examples of the backgrounds at each site.

There were clear differences in the substrate composition of each of our four sites (Fig. 4). For Falmouth, the upper and middle shore heights were largely made of rock and gravel/stone substrate, illustrating the predominant rockpool environment. Moving down the shore, the amount of green and brown algae/seaweed substantially increases. There is red-encrusting algae at all zones, but especially the upper and middle zones. St Mawes is also a predominantly rockpool habitat, illustrated by the fact that the site is dominated by rock and gravel at all three zones. As with Falmouth, there is an increase in red and green algae lower down the shore. Although St Mawes is less diverse in terms of substrate types than Falmouth, note that our surveys here do not account for how visually diverse the substrate colors and contrasts are, or their spatial scale, within a given substrate category. Subjectively, St Mawes is highly variable in substrate colors in terms of the rock and gravel present.

In contrast to Falmouth and St Mawes, Godrevy is a site dominated by mussel beds and a rocky substrate. There are few changes with shore height in substrate proportions. Helford, a site we have classified as mainly mudflats, shows clear changes in substrate type with shore height. At upper shore heights, the site is mainly composed of rocky substrate and gravel. Moving down the shore the substrate changes to become dominated by mud/sediment, with some green/brown algae and gravel. Note, however, that despite the predominantly rockpool nature of the upper shore, these rockpools contained large amounts of mud and sediment, such that the appearance of the background at all three shore heights was relatively similar (muddy).

### Differences in appearance between life stage, site, and shore height

**Figure 5:**
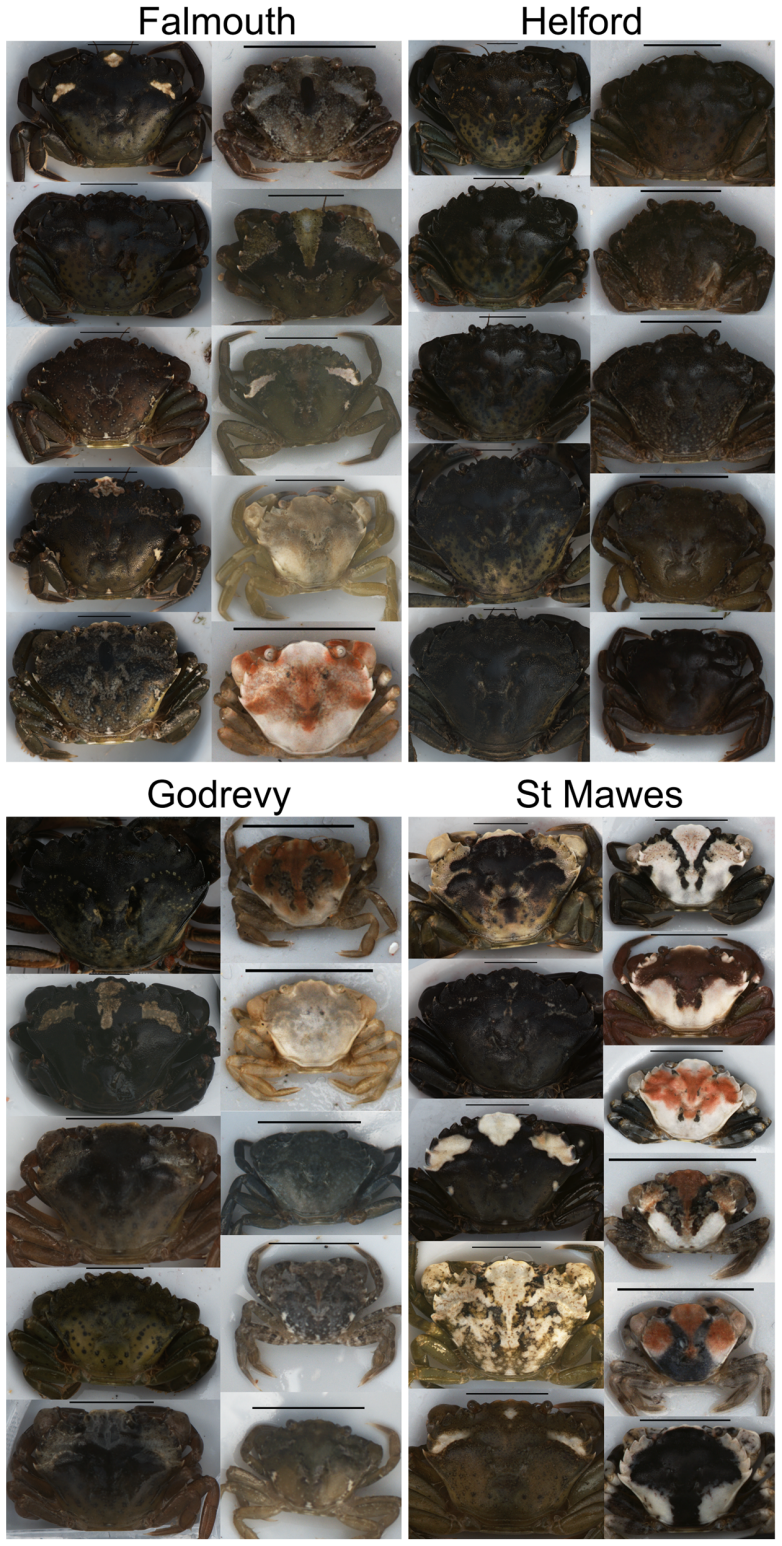
Difference in the appearance of adult and juvenile crabs from the four study sites. Each panel shows crab phenotypes that to human eyes were broadly most representative of the common appearances found at each site. In each case, adult crabs are in the left columns and juveniles on the right. Broadly, crabs from St Mawes were the most diverse (hence the extra numbers of crabs shown here), in addition to juvenile crabs from Falmouth. Both these crab types show high diversity in color and pattern. In contrast, crabs from Helford were least diverse and were more uniform (less patterned) and blue-green in color.

**Figure 6:**
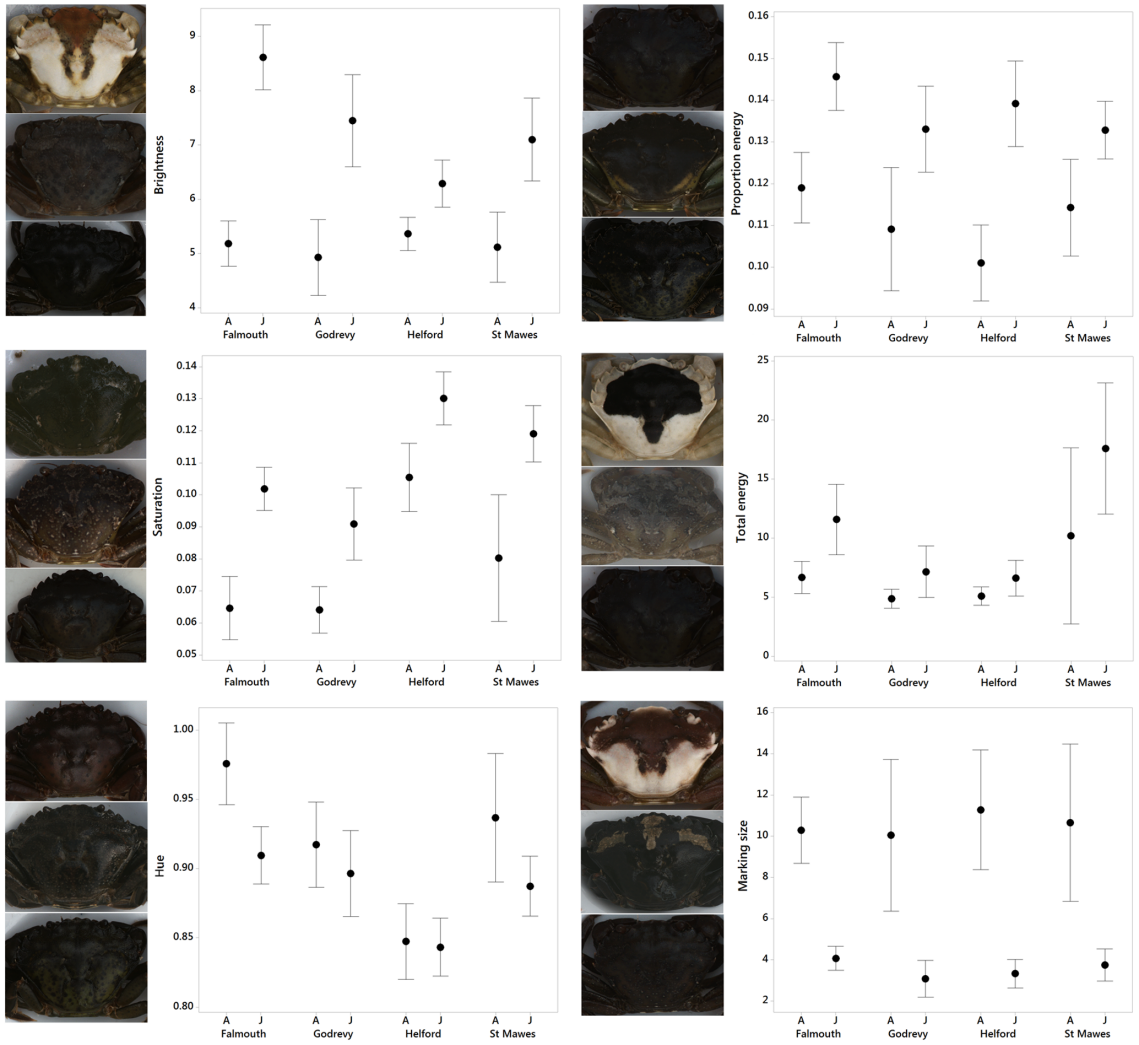
Differences in the appearance of adult and juvenile crabs from four different sites. Crabs show a wide range of significant differences among sites and life stages. Brightness is the overall reflectance of the carapace, saturation can be considered as the amount of a given color type compared to white light or color richness, and hue is the color type and is based on a ratio of color channel values (see main text), with lower values meaning that crabs are more blue-green in color. Proportion energy relates to how much one marking size dominates the crab patterns (larger values mean one or a few markings are prevalent), total energy equates to the contrast of the patterns (higher values mean more contrasting markings), and marking size is the predominant marking size found on the crabs. Small thumbnail images of crabs correspond to example individuals from the entire dataset (across all sites and life stages) with the maximum (top images), minimum (bottom images), and approximately average (middle images) values for each appearance metric.

**Figure 7:**
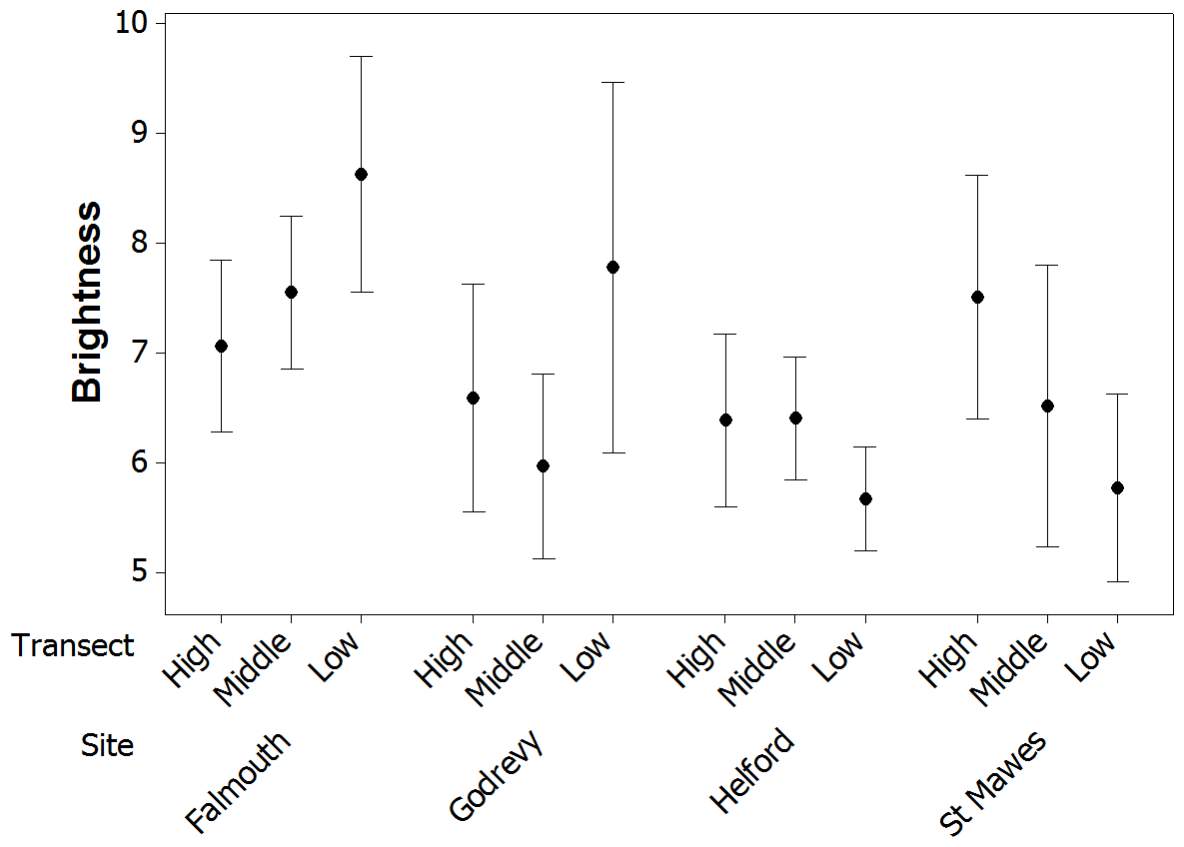
Differences in the brightness of crabs among sites at different heights of the shore. As well as showing significant differences in brightness (overall carapace reflectance) among sites, there were clear effects of shore height at Falmouth and St Mawes. At the former, crabs are brighter lower down the shore, whereas at the latter there is the opposite relationship. Crabs from Godrevy and Helford show little relationship between brightness and shore height.

Crabs showed clear differences in appearance among sites and life stages (Fig. 5), and there were significant differences in our General Linear Models (GLMs) in appearance for crabs from different sites and of different ages (Fig. 6). For brightness, data was Box Cox transformed (power of −1.124). There was a significant interaction between site and life stage (F = 4.33, df = 3,661, p = 0.004), and between site and zone (F = 3.34, df = 6,661, p = 0.003), with significant effects in the model of site (F_(3,661)_  = 3.11, p = 0.026), life stage (F = 67.86, df = 1,661, p<0.001), but not zone (F = 0.53, df = 2,661, p = 0.591). Generally, juveniles have higher brightness values than adults, but the magnitude of this difference depends on the site, with particularly large differences in brightness between adults and juveniles at Falmouth and Godrevy. In general, crabs from Helford were darker, and with small differences between adults and juveniles. Interestingly, crabs from Falmouth are brighter lower down the shore, whereas crabs from St Mawes become darker (Fig. 7). Crabs from Godrevy and Helford show fewer changes with zonation, perhaps because these habitats vary less in appearance at different heights.

For saturation, data was Box Cox transformed (power of 0.562) and there were significant effects in the model of site (F = 23.25, df = 3,661, p<0.001), life stage (F = 68.63, df = 1,661, p<0.001), but not zone (F = 0.16, df = 2,661, p = 0.855). There were no significant interactions. Adults have lower saturation values than juveniles. Crabs from Helford are generally more saturated than crabs from other sites, though juveniles from St Mawes also show high saturation values. Adult crabs from Falmouth and Godrevy have lowest saturation values.

For hue there were significant effects in the model of site (F = 14.19, df = 3,661, p<0.001), life stage (F = 12.93, df = 1,661, p<0.001), but not zone (F = 2.37, df = 2,661, p = 0.094). There were no significant interactions. Crabs from Helford have the lowest hue values, which equates to more blue-green coloration. Crabs from other sites have coloration closer to grey (more red-brown), but note that much of the color differences would also occur in the color channel hue 1, which we did not analyze further due to the correlation with saturation (see Methods).

**Figure 8:**
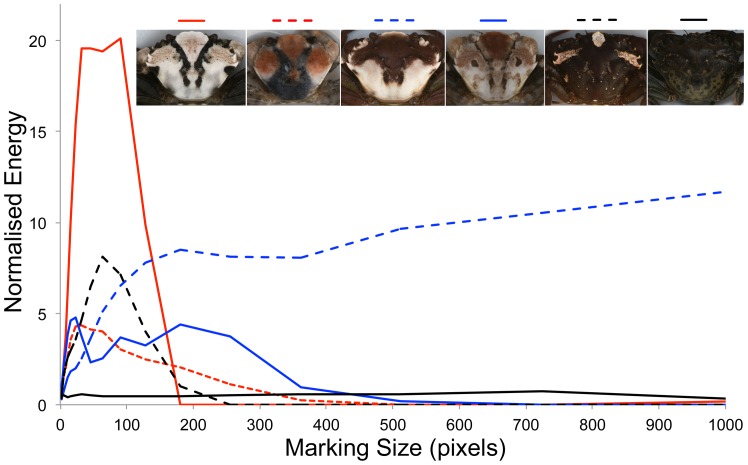
Example granularity spectra used to calculate aspects of crab patterns. There were large differences in the patterns of crabs with some showing markings dominated by a small number of pattern sizes of very high contrast (spectra with strong peaks), and others with more uniform appearances with a range of low contrast patterns (relatively flat spectra of low amplitude). There were strong associations of pattern attributes with different sites.

There were substantial differences in pattern among crabs, demonstrated by the diversity of granularity spectra obtained (Fig. 8). Data for proportion energy was Box Cox transformed (power of −0.450). There were significant effects in the model of site (F = 2.75, df = 3,661, p = 0.042), life stage (F = 62.36, df = 1,661, p<0.001), but not zone (F = 0.30, df = 2,661, p = 0.743). There were no significant interactions. Juvenile crabs have higher proportion energy values (with little differences among sites), meaning that their markings are more dominated by one of a few relatively pronounced patterns. Adult crabs from Godrevy and Helford had low proportion energy values compared to crabs from Falmouth and St Mawes.

For total energy, the data was log transformed. There was a significant effect in the model for only site (F = 5.64, df = 3,661, p = 0.001), but not life stage (F = 1.30, df = 1,661, p = 0.255), or zone (F = 0.66, df = 2,661, p = 0.518). There were no significant interactions. Crabs from St Mawes had higher total energy values (more contrasting markings), whereas crabs from Godrevy and Helford had relatively low values.

Marking size data was Box Cox transformed (power of 0.113). There was a significant effect in the model of life stage (F = 103.77, df = 1,661, p<0.001), but not zone (F = 1.41, df = 2,661, p = 0.245). There was only a non-significant trend for differences among sites (F = 2.41, df = 3,661, p = 0.066). Here, larger crabs had bigger markings, as would be expected with the increase in body size.

### Differences in size with site and shore height

We tested whether there was a difference in the size of crabs among sites and between the three heights on the beach with a GLM (data log transformed), with carapace width as the response and site and zone in the model. There was no difference between the size of crabs for either site (F = 0.48, df = 3,671, p = 0.694), or zone (F = 2.20, df = 2,671, p = 0.111), nor was there a significant interaction between site and zone.

### Distinctiveness of crabs among sites

We conducted DFA on the appearances of crabs among sites for both adults and juveniles based on the metrics of color and pattern (Table 1). Juvenile crabs were correctly assigned at a proportion of 0.435, and there were differences in the level overlap among categories (sites), with crabs from Godrevy being put most often into the wrong group, and crabs from Helford and Falmouth being most identifiable. As would be expected if crabs take on the appearance of their habitat as they molt, adults were correctly assigned to their own site more often than juveniles: 0.538 proportion of times. Crabs from Helford and Falmouth were correctly assigned at a proportion above 0.6. However, crabs from St Mawes and Godrevy were correctly assigned less than 0.18 proportion of times, implying that these crabs have strong features in common with all sites and less distinctiveness.

**Table 1:** Results of the discriminant function analysis, classifying crabs into their respective sites based on the color, brightness, and pattern metrics.

	Juveniles				Adults			
Site	Falmouth	Godrevy	Helford	St. Mawes	Falmouth	Godrevy	Helford	St. Mawes
Falmouth	**147**	43	53	63	**60**	19	16	17
Godrevy	0	**1**	0	0	3	**5**	0	2
Helford	24	7	**40**	40	6	5	**26**	8
St. Mawes	22	12	23	**33**	2	0	0	**0**
Total N	193	63	116	136	71	29	42	27
**Proportion Correct**	**0.762**	**0.002**	**0.345**	**0.243**	**0.845**	**0.172**	**0.619**	**0.000**

For both juveniles and adults, crabs from Falmouth (mainly rockpool) and Helford (mainly mudflat) are classified with high fidelity. In contrast, crabs from Godrevy (mussel bed) and St Mawes (rockpools) are classified with much less accuracy.

### Level of individual variation among and within sites and age classes

**Figure 9:**
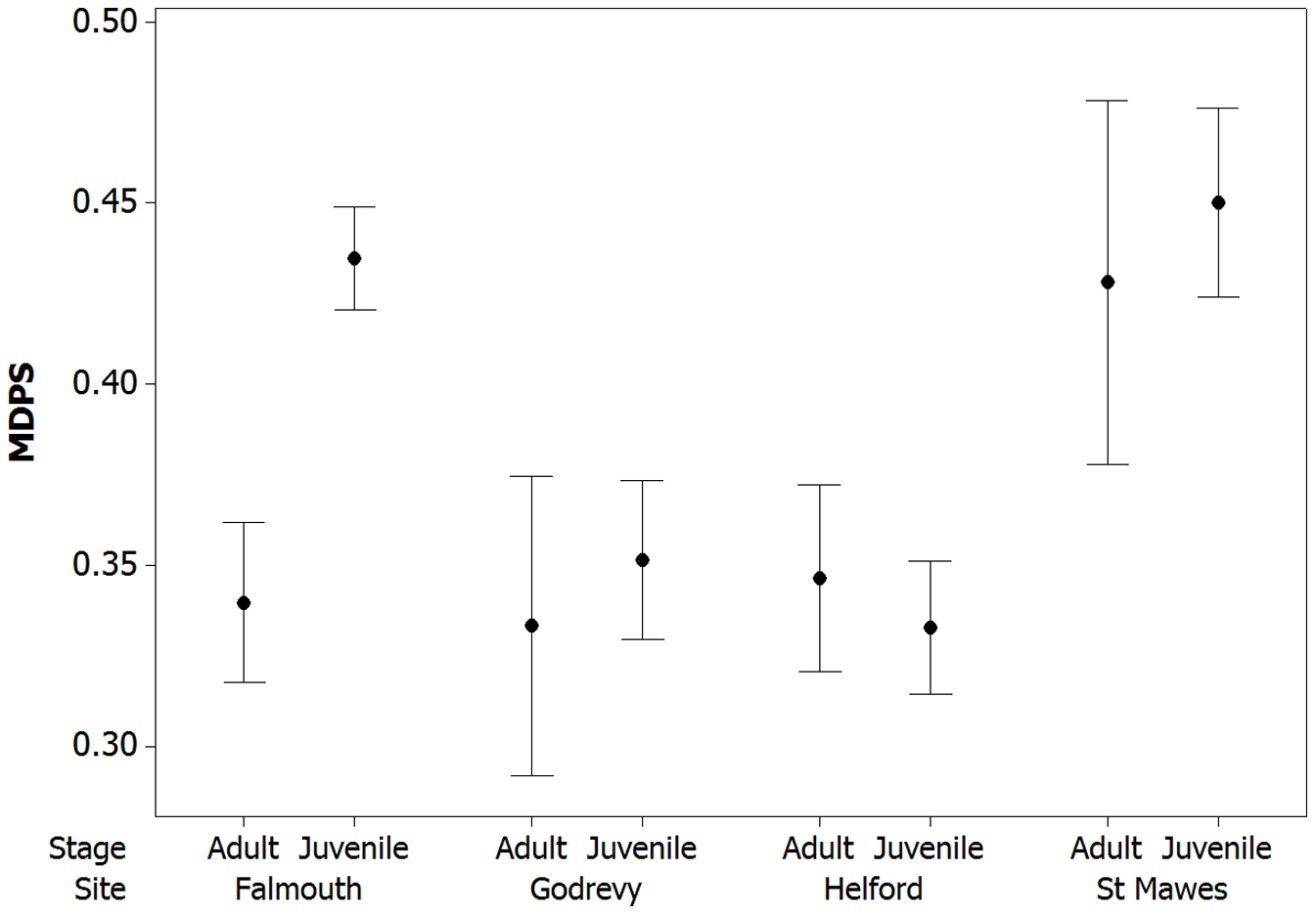
Differences in the individual diversity of crab appearances among sites and ages. Data were analyzed in a multidimensional phenotypic space (MDPS) incorporating all six measurements of color and pattern (see main text). Crabs from St Mawes and juveniles from Falmouth show very high levels of individual variation, whereas crabs from other sites were less variable and showed little difference between adults and juveniles.

We compared whether crabs from different sites are more or less variable in a multidimensional phenotypic space (MDPS), and this revealed some clear differences in the individual diversity of crabs from different sites and age categories (Fig. 9). The data was transformed via Box-Cox transformation (power of −1.57) followed by being squared. We ran a GLM with MDPS values as the response and site, life stage, and an interaction between site and life stage included in the model. There were significant effects of all three: site-stage interaction (F = 31.14, df = 3,676, p<0.001), site (F = 37.15, df = 3,676, p<0.001), and stage (F = 14.94, df = 1,676, P<0.001). For juveniles, mean variation was as follows: Falmouth (0.435, resampled  = 0.428+/−0.022 StD), Godrevy (0.351), Helford (0.333, resampled  = 0.329+/−0.018 StD), and St Mawes (0.447, resampled  = 0.442+/−0.028 StD). Thus, there was relatively little change to the MDPS estimates with the resampled values down to the lowest sample size (Godrevy, 63). For adults, mean variation was as follows: Falmouth (0.339, resampled  = 0.327+/−0.027 StD), Godrevy (0.333, resampled  = 0.321+/−0.011 StD), Helford (0.346, resampled  = 0.329+/−0.019 StD), and St Mawes (0.428). Again, there was relatively little change to this with the resampled values down to the lowest sample size (St Mawes, 29). Thus, both adults and juveniles from St Mawes and juveniles from Falmouth had high levels of variation, whereas levels of variation were low for all crabs from Godrevy and Helford, and for adults from Falmouth.

## Discussion

Here, we tested for phenotype-environment associations in the shore crab among four sites of different habitat composition, and for differences between adults and juveniles. First, we found significant differences in the appearance of crabs among each site for color, brightness and pattern. Adult and juvenile crabs showed clear differences, with adult crabs generally darker and less saturated in coloration than juveniles. Adults also had less contrasting or prominent markings, and larger making sizes. However, these differences sometimes depended on the site. We also found corresponding changes in appearance associated with continuous increase in crab size. Furthermore, as crabs get larger the spread of values in the correlations decreases for many of these appearance metrics, reflecting the fact that juvenile crabs show greater variation. These differences with size/age also translate into a high level of distinctiveness between adult and juvenile crabs. Our discriminant function analysis (DFA) showed that approximately 84% of crabs are correctly classified as either adult or juvenile based on our appearance metrics. Differences in appearance between adult and juvenile crabs, and specifically less diversity in adult crabs, have been widely reported before (e.g. [31], [33], [34], [63]), but ours is the first study to directly quantify this.

Regarding differences among sites, crabs from Helford, a site characterized by relatively dark green-brown mudflats and sediment, were generally darker than crabs from other sites. There was little change in brightness with zonation, perhaps because this habitat varies less in appearance overall, which is consistent with our observations that even the rockpools on the upper shore contained a great deal of mud, even though the background types did vary with zone. Crabs had lower hue values at Helford than elsewhere, meaning that they were more blue-green in coloration, and they had relatively high saturation values meaning that their color was richer or less achromatic. Helford crabs also tended to have relatively low total energy values, illustrating that their patterns were not very prominent, again consistent with the more homogeneous mud background. In contrast, crabs from Falmouth (mainly rockpools) are brighter lower down the shore than higher up. This may be because higher up the shore has more dark rock, whereas lower down the shore there is a greater amount of green and brown algae and other changes in substrate, which may be brighter in coloration or allow different types of camouflage. There are also considerable differences in the brightness of adults and juveniles, with juveniles having higher reflectance and also being brighter than crabs from other sites. Both adult and juvenile crabs from Falmouth have relatively high proportion energy and total energy values, indicating that they have relatively prominent markings. This is in accordance with the relatively high-contrast backgrounds found at this site, with lots of rocks and substrate of different colors and of varied grain size, and the wide variety of background substrate types (Fig. 4). Crabs from St Mawes (also mainly rockpools) become darker lower down the shore, which is consistent with our habitat transects which showed an increase (albeit not a dramatic one) in the amount of dark rock lower down the shore. Juveniles in particular also have high saturation values compared to most other sites. Otherwise, both adults and juveniles had clearly the highest values for total energy across sites, showing that their patterns are of especially high contrast. Finally, crabs from Godrevy (mainly mussel beds) show few changes in brightness with zonation, consistent with the fact that this habitat varies little in substrate appearance with shore height. Indeed, our habitat surveys showed little change in the proportion of substrate types with shore height, with all three zones dominated by mussel bed and rock. Adult crabs from Godrevy are relatively dark compared to crabs from other sites, whereas juveniles are relatively bright. Godrevy crabs generally have low saturation values and adults are relatively blue-green in coloration. Otherwise, they have relatively low total energy values, indicating that their markings are not very prominent.

We also used DFA to determine how distinct crabs from each site are in overall appearance. The results showed that for both juveniles and adults, 43–53% of individuals are correctly classified to their respective sites. Although this is not especially high (approximately half of the individuals were correctly assigned), given that there were four sites studied and that the sites overlap to a lesser or greater extent in habitat substrate (Fig. 4), this level of classification suggests that crabs from each location show reasonable levels of distinctiveness. However, the level of distinctiveness depends on the location. Crabs from Helford and Falmouth are correctly classified between 34% and 85% of the time, which is in accordance with the relatively distinctive nature of the backgrounds at Helford and characteristic substrate colors and patterns at Falmouth. In contrast, crabs from Godrevy and St Mawes are correctly classified between only 0% and 24% of the time, which fits with St Mawes at least having a wide range of substrate types and visual appearance.

In terms of the level of individual variation, crabs from St Mawes (both adults and juveniles) were highly variable in the multidimensional phenotypic space (MDPS). This is likely to stem from the (subjectively) considerable variation in background appearances at this location, although the site did not have a large range of substrate types present. In contrast, crabs from Godrevy and Helford, as well as adult crabs from Falmouth, had relatively low MDPS values. For Godrevy and Helford, this makes sense because the mussel bed and mudflat backgrounds at these locations are relatively uniform in appearance (especially the mudflats). At Falmouth, juvenile crabs, however, were highly variable. This difference is intriguing and we think it most likely reflects differences in matching to the substrate scale with size (see below), perhaps also linked to the wide range of substrate types present.

Overall, we have shown clear differences in the appearance of crabs among sites. In addition, we also show how variation in appearance among sites is often quite different between adults and juveniles, and find clear differences in the amount of individual diversity associated with site. While other studies have investigated phenotype-environment associations in shore crabs before [33], [63], most notably the excellent work by Todd et al. [34], [35], our study has made substantial progress beyond these. Previous studies investigated crab appearance with human vision, which is known to be inappropriate to judge the colors of animals where humans are not the natural receivers (e.g. predators [4], [64]), and is incapable of quantifying specific features of appearance. Second, previous work grouped crabs into a number of subjectively defined categories of appearance (‘morphs’), yet it is unclear whether shore crabs do exist in discrete morphs, as opposed to exhibiting continuous variation. In contrast, analyzing specific metrics of appearance, as we have done here, allows an analysis of how aspects of appearance vary with habitat (for example, marking size). Third, past work did not distinguish or directly compare adults and juveniles, nor did it analyze and quantify levels of individual variation, and how this is related to habitat type/location. Our work has addressed all these above issues and more, making it the most comprehensive study of this subject undertaken to date. Nonetheless, we note that Todd et al. [35] found that phenotype-environment associations between patterned and unpatterned crabs were strongest at a small (m^2^) scale, something which we did not investigate here, and this would be important to further investigate in future work.

Our results demonstrate clear differences in crab appearance associated with site/habitat. It should be noted, however, that as with past work we have not shown a direct link to camouflage. This requires demonstrating that differences in the appearance of crabs among sites translates into improved camouflage at their own location versus other locations; that is, phenotype-environment matching. It will be important to do this in future work. However, this is non-trivial for several reasons. First, the range of backgrounds encountered, with many being underwater or partially submerged, makes quantifying the background challenging. We were unable to accurately quantify background appearances in the current work, although this is a major future aim of ours. Second, camouflage needs to be analyzed to predator vision, yet crabs are attacked by a wide range of species, especially various bird and fish [42]. They are likely to be attacked by both gulls and other types of shore bird, and while gulls appear to have a visual system well tuned to ultraviolet wavelengths (a ‘UVS’ system), other shore birds seem to have a violet (‘VS’) system, still sensitive to UV light but shifted to longer wavelengths in terms of their UV cone type [65]. The main fish predators of crabs are likely to mainly comprise dichromatic teleosts, such as pollack (*Pollachius pollacbius* [66]), but also dogfish sharks (*Scyliorhinus canicula*), which appear to be monocromats [67]. Therefore, quantifying the camouflage of shore crabs would involve modeling four different types of visual system, some of which may be more important in driving camouflage at different age classes. Nonetheless, we aim to quantify camouflage to predator vision in a future study, and to undertake experiments to test how this affects detection probability.

Regardless of the lack of direct quantification of camouflage (which applies to most if not all studies of phenotype-environment associations to date), we believe that camouflage is by far the most likely explanation for our results. First, crab differences in appearance with habitat (and for brightness with shore height too) cannot stem from differences in size since we found no difference in the sizes of crabs among sites/zones. In addition, as Todd et al. [34] argue, the existence of such clear associations between crabs and substrate type, as well as the likely mixing of crabs among substrates/sites (shore crabs can be highly mobile [68]), makes UV protection, thermoregulation, and diet unlikely explanations for carapace patterns. Furthermore, our sites are all in a relatively small geographical area, meaning that there would likely be relatively few differences in light radiation, water temperature and salinity (although microclimates will undoubtedly exist to some extent).

The presence of phenotype-environment associations in this and previous work raises the question of what mechanisms drive it. As Todd et al. [35] have outlined, the mechanisms involved are likely to differ based on timescale and spatial scale. Matching at the micro scale and over short time periods (ca. minutes to days and at a 1 m^2^ scale) is likely to be driven by differential predation on mismatched crabs, short term color change, and behavioral choice of substrates, at the meso-scale and medium time periods (ca. weeks and months and 100 s–1000 s m^2^) by longer term color change and phenotypic plasticity through molting, and at the macro-scale and over longer term (years and 10,000 s m^2^) by differential predation and genetic adaptation.

Like many other animals [69], [70] shore crabs are able to change brightness depending on the appearance of the background over a period of less than two hours [45], [63]. However, it is unlikely that the results in our study were due to color change over a period of a few hours because crabs were collected first and kept in a grey or white bucket before photography, and changes in coloration reported so far are relatively small and likely to be restricted to juveniles [45]. Color change over a period of days and weeks due to redistribution of pigment cells is perhaps more plausible and there is some evidence that this may occur in shore crabs [32], although this remains to be fully tested. It is also widely assumed that crabs change appearance with molting through phenotypic plasticity (e.g. [33], [34], [42]), and this is perhaps the most likely explanation in driving some of the larger differences in appearance among site and age/size that we find here. Hogarth [33] reports in crabs that were allowed to molt small changes in pattern in about 30% of crabs, and major changes in pattern in about 10% of individuals. However, Hogarth presents no information on the number of crabs tested or what backgrounds they were placed on. Here, we found that adult crabs from different sites were more distinctive in being classified into the correct group than were juvenile crabs. Again, although indirect, this is consistent with the hypothesis that crabs gradually adopt the appearance of each site as they molt.

Variation and matching among sites could also arise if populations evolve genetic differences, and there is some evidence for distinct genetic populations of shore crabs across large geographic regions of Europe [71], [72]. Within the UK, morphological variability in size and shape has been detected in crabs from different locations, and that overall around 20% of the phenotypic variability detected was associated with patterns of genetic similarity [73]. In sites in Devon and Cornwall, variation at eight microsatellite loci suggested that the South West UK comprises a single population of shore crabs, but there may sometimes be a small degree of isolation of subpopulations [74]. However, due to a planktonic larval stage, large genetic differences among geographically close sites are generally unlikely [33]. Thus, while there is the potential for our results to be driven by genetic adaptation, this is unlikely here given the close proximity of sites.

Finally, it is possible that crabs are able to behaviorally choose backgrounds that improve their camouflage. Indeed, we have found some preliminary evidence that adult crabs at least will choose between substrates of different appearance (unpublished data). This mechanism may underlie associations at the micro scale, such as with different shore heights. Camouflage based on behavioral choice is likely to be more common than previously thought across a wide range of species, and there is growing evidence that some animals select backgrounds and resting postures to improve their match to the background, including some birds and moths (e.g. [75], [76]). Behavioral choice need not just involve choosing specific local patches, but could also operate at a larger scale involving general choice of habitat, especially given that shore crabs are known to sometimes move between 0.5–2 km in a short space of time [68]. For example, the limpet *Lottia digitalis* has camouflaged shell colors that vary in appearance. Byers [77] found that the shell is typically dark brown or grey when individuals are found on rock surfaces but white with black lines when individuals occur with barnacles. He marked individuals and moved them to new locations, and found over a 12-day period that limpets moved back to their original habitat types (rather than homing back to their exact original locations), showing a habitat specific preference linked to appearance. Overall, we feel that behavioral choice, combined with phenotypic plasticity and longer term color change, are the most likely explanations for our findings.

The question of why crabs change appearance as they grow and become less patterned with age is somewhat of a mystery, although there is range of potential explanations, many of which are not mutually exclusive. First, smaller shore crabs may be associated with different substrate types [33]–[35], and juvenile forms may differ from adults if the backgrounds where they are found also differ [32], [34], [46]. Second, crabs may potentially lose patterns with age if changes in size make the patterns more conspicuous if they begin to deviate from the background features in the environment [33], [34]. Third, juvenile crabs may be especially vulnerable to predation, perhaps being targeted by both birds and fish, and so may need more effective camouflage [31]. Different sized crabs may also vary in their risk of predation due to behavioral differences, such as amount of time spent foraging and actively defending themselves when attacked [33]. Fourth, adults may be more uniform if many juvenile forms are actually not well matched to the background and are then selectively removed by predation with age [33]. Finally, high levels of individual variation may prevent predators from forming a search image, whereby predators show an increase in their ability to find a familiar prey type across successive encounters and a corresponding reduction in ability to find less common prey. Search image formation can result in predators finding a disproportionate amount of more common prey types, leading to negative-frequency dependent selection, whereby rare forms are at a selective advantage [12], [39]. Further work analyzing the properties of the different substrates crabs are found on and the distribution of phenotypes are needed to answer these questions.

Phenotype environment matching and camouflage has the potential to help us to understand how animals adapt to different environments and the selection pressures that produce this. In addition, the subject can be a valuable means to explore the mechanisms that produce environment associations, and what drives individual variation and diversity in general, and their relative importance. Crabs are an especially valuable group to utilize because they often suffer considerable predation pressure and show a wide range of adaptations for camouflage [34], [50], [78], [79], and it is likely that multiple mechanisms underlie this in different habitats, such as genetic adaptation among populations, phenotypic plasticity, color change, and behavioral strategies and choices. There is much left to discover about camouflage and how it works, especially in real species, and in understanding how camouflage evolves in different habitats. Future work needs to more rigorously quantify the appearance of animals and the backgrounds on which they are found, and to analyze camouflage matching in terms of the visual system of the predators involved.

## Supporting Information

S1 Data **Experimental data.**
(XLSX)
